# Practical Remarks on Some Exhausting Diseases, Particularly Those Incident to Women

**Published:** 1845-07

**Authors:** 


ART. III.—*Practical Remarks on some exhausting Diseases, particularly those incident to Women.* By SIR JAMES EYRE, M.D.—*London,* 1845. 8vo, pp. 76.

THE object of this tiny volume, which it would be somewhat difficult to gather from its title, is to recommend the *oxide of silver*—originally introduced some years since into practice by Mr. Lane—"in pyrosis, in certain cases of gastric disorder, in the slowly exhausting hemorrhage from mucous surfaces, but, above all, in atonic menorrhagia, which, though arising from various causes, and hence often most perplexing to the practitioner, will, it is confidently predicted, become henceforth as amenable to treatment as it has been hitherto unmanageable." (p. 1.)

The dose in which Sir James Eyre employs this remedy never exceeds three grains daily, and he usually commences with quarter or half grain doses thrice a day. The existence of febrile action in any considerable degree, is the only circumstance that contraindicates its employment, and though he disclaims for it the character of a specific, yet the detail of thirty-nine successful cases, and no mention of its failure in a single instance, would seem almost to have warranted his claiming for it that title. For ourselves, we are much too old, and we should have thought Sir James not young enough to be seduced, by the successful issue of a few cases, into the somewhat juvenile beatitude of indulging hopes of this sort. The oxide of silver may be deserving a place among the thousand remedies that distract doctors; we do not say that it may not yet be proved to be a truly valuable therapeutic agent: on Sir James's authority, and on Mr. Lane's, we recommend our readers to give it a trial,—a fair trial; but our worthy knight must excuse us for saying that there is scarcely one medicament in our boundless store, however much scorned now, that has not in its day had as good (or better) evidence of its power and value, as he has adduced in favour of his oxide. It appears, also, on the authority of Mr. Lane himself, that this preparation, contrary to what was expected and believed, does occasionally blacken the skin like the nitrate; and while such a terrible chance overhangs its employment,—we should have unquestionable evidence of its superiority to less deleterious agents, before we accept it as a medicine of ordinary use. What volumes have been written on the vast powers of the nitrate of silver, in curing epilepsy, gastralgia, &c. &c.! And yet we are not afraid to say that the positive facts of some dozens of real men and women, existing within this realm, silvered into an everlasting blueness, are evils of a magnitude to counterbalance, a hundred fold, all the demonstrable good that was ever produced by all the caustic that was ever swallowed.

In the brief statement given above, our readers are presented with the whole sum and substance of Sir James Eyre's observations; for the details of the cases are too meager to throw any fresh light on the diseases which they are intended to illustrate, and the practical remarks consist of quotations from 'Copland's Dictionary,' the 'Medico-Chirurgical Review,' &c. &c. which answer no very obvious purpose except that of making the little book bigger.

The results of Sir J. Eyre's experience would have become much more extensively known, if he had embodied them in the form of a brief communication to one of the weekly medical journals; and we cannot understand the strange error of judgment into which he has fallen, in supposing that he has chosen a mode of announcing them either "more comprehensive," or "more convenient," than such a vehicle would have supplied. This, however, is more Sir James's affair than ours; and we must not conclude without qualifying what we have already said by this additional remark, that the profession is under obligations to any of its members, who carefully and faithfully exerts himself to improve the therapeutical part of the art; and this our author has certainly done.

